# Non-sedating benzodiazepines cause paralysis and tissue damage in the parasitic blood fluke *Schistosoma mansoni*

**DOI:** 10.1371/journal.pntd.0007826

**Published:** 2019-11-15

**Authors:** Paul McCusker, Md Yeunus Mian, Guanguan Li, Michael D. Olp, V. V. N. Phani Babu Tiruveedhula, Farjana Rashid, Lalit Kumar Golani, Ranjit S. Verma, Brian C. Smith, James M. Cook, John D. Chan

**Affiliations:** 1 Department of Cell Biology, Neurobiology & Anatomy, Medical College of Wisconsin, Milwaukee, WI, United States of America; 2 Department of Chemistry and Biochemistry, Milwaukee Institute of Drug Discovery, University of Wisconsin-Milwaukee, Milwaukee, WI, United States of America; 3 Department of Biochemistry, Program in Chemical Biology, Medical College of Wisconsin, Milwaukee, WI, United States of America; 4 Department of Pharmacology and Toxicology, Medical College of Wisconsin, Milwaukee, WI, United States of America; Consejo Nacional de Investigaciones Cientificas y Tecnicas, Fundación Mundo Sano, ARGENTINA

## Abstract

Parasitic flatworm infections (e.g. tapeworms and fluke worms) are treated by a limited number of drugs. In most cases, control is reliant upon praziquantel (PZQ) monotherapy. However, PZQ is ineffective against sexually immature parasites, and there have also been several concerning reports on cestode and trematode infections with poor PZQ cure-rates, emphasizing the need for alternative therapies to treat these infections. We have revisited a series of benzodiazepines given the anti-schistosomal activity of meclonazepam (MCLZ). MCLZ was discovered in the 1970’s but was not brought to market due to dose-limiting sedative side effects. However, in the decades since there have been advances in our understanding of the benzodiazepine GABA_A_ receptor sub-types that drive sedation and the development of sub-type selective, non-sedating ligands. Additionally, the sequencing of flatworm genomes reveals that parasitic trematodes and cestodes have lost GABA_A_R-like ligand gated anion channels, indicating that MCLZ’s anti-parasitic target is distinct from the human receptors that drive sedation. Therefore, we have screened a library of classical and non-sedating 1,4-benzodiazepines against *Schistosoma mansoni* and identified a series of imidazobenzodiazepines that immobilize worms *in vitro*. One of these hits, Xhe-II-048 also disrupted the parasite tegument, resulting in extensive vacuole formation beneath the apical membrane. The hit compound series identified has a dramatically lower (~1000×) affinity for the human central benzodiazepine binding site and is a promising starting point for the development of novel anti-schistosomal benzodiazepines with minimal host side-effects.

## Author summary

Over 200 million people are infected with schistosomiasis, yet there are limited therapeutic options available to treat this disease. The benzodiazepine meclonazepam is known to cure both intestinal and urinary schistosomiasis in animal and human studies, but dose-limiting sedation has been a barrier to its development. Little is known about the structure-activity relationship of meclonazepam and other benzodiazepines on schistosomes, or the identity of the parasite receptor for these compounds. However, schistosomes lack obvious homologs to the human GABA_A_Rs that cause sedation. This indicates that the parasite target of this drug is distinct from the host receptors that underpin dose-limiting side effects of meclonazepam and raises the possibility that benzodiazepines with poor GABA_A_R affinity may still retain anti-parasitic effects. Here, we report an *in vitro* screen of various benzodiazepines against schistosomes, and the identification of hit compounds that are active against worms yet display reduced affinity for the human GABA_A_R that causes sedation.

## Introduction

Over 200 million people are infected with the parasitic blood flukes that cause the neglected tropical disease schistosomiasis [1], and over 90% of infections occur in sub-Saharan Africa where the disease kills approximately 280,000 persons/year [2, 3]. Chronic infection adds to the disease burden, putting the socioeconomic cost of schistosomiasis (70 million disability adjusted life years [4]) near HIV/AIDS, malaria or tuberculosis [1]. However, despite these enormous costs treatment relies on just one broad-spectrum drug, praziquantel (PZQ) [5]. PZQ treatment has high cure rates of 70–90% [6, 7], but it is concerning that a subset of infections in human and animal populations appear to be refractory to treatment [8–10], either due to PZQ’s lack of efficacy against recently acquired, immature parasites [11, 12] or standing genetic variation in parasite populations. The latter possibility is especially concerning in regard to the potential emergence of PZQ-resistant parasites, and consideration needs to be given to whether PZQ-monotherapy will be sufficient to achieve schistosomiasis elimination [13].

One lead compound with proven anti-schistosomal activity is the benzodiazepine meclonazepam ((S)-3-methylclonazepam, MCLZ). MCLZ was discovered in the 1970’s and found to cure both the mature and immature parasites that cause urinary and intestinal forms of schistosomiasis [14]. Development of this lead stalled in the 1980’s due to dose-limiting sedation in human trials [15–18]. However, we have re-visited benzodiazepines as potential anti-parasitic leads given advances in two areas. First, it is now understood that the sedative effects of benzodiazepines are driven by GABA_A_Rs that contain the α1 subunit [19]. This has enabled the design of various benzodiazepines with reduced affinity towards α1-containing GABA_A_Rs to treat conditions such as asthma and schizophrenia that involve GABA_A_Rs that contain other α subunits [20]. Second, with recent advances in the sequencing of parasitic helminth genomes there is an abundance of data available to establish whether flatworm parasites possess GABA_A_Rs [21]. If GABA_A_Rs are not present in parasitic worms, then it is possible that the structure-activity requirements of anti-parasitic compounds may differ significantly from those mediating benzodiazepine binding to host GABA_A_Rs, offering the opportunity to develop ligands with increased parasite selectivity. Here, we have profiled the repertoire of *S*. *mansoni* ligand gated ion channels and, having found no obvious parasite GABA_A_Rs, screened a library of benzodiazepines to identify compounds that display anti-parasitic activity and exhibit reduced mammalian GABA_A_R affinity.

## Materials and methods

### Ethics statement

Animal work was carried out with the oversight and approval of the Laboratory Animal Resources facility at the Medical College of Wisconsin, adhering to the humane standards for the health and welfare of animals used for biomedical purposes defined by the Animal Welfare Act and the Health Research Extension Act. Experiments were approved by the Medical College of Wisconsin IACUC committee (approved protocol #AUA00006471 and AUA00006735).

### Bioinformatic prediction of flatworm ligand-gated ion channels

Putative ligand-gated ion channels were curated from a diversity of organisms sampling vertebrates (human), arthropods (*Drosophila melanogaster*), nematodes (*Caenorhabditis elegans*), and mollusks (*Aplysia californica*). The predicted proteomes of these organisms were searched for gene products containing a ligand-gated ion channel (LGIC) Pfam domain (PF02932). These were used to generate hidden Markov probability models (HMMER v3.2.1), which were used to search the predicted proteomes of various free-living *(Schmidtea mediterranea—PRJNA379262*, *Macrostomum lignano—PRJNA371498*) and parasitic (*Schistosoma mansoni—PRJEA36577*, *Schistosoma haematobium—PRJNA78265*, *Clonorchis sinensis—PRJNA386618*, *Opisthorchis viverrini—PRJNA222628*, *Echinococcus multilocularis—PRJEB122*) flatworms for putative LGICs. Resulting candidates were filtered based on number of predicted transmembrane domains (TOPCONS predictions of between 3–5 transmembrane domains) [22]. The retained sequences were then aligned (Clustal Omega), manually inspected for the presence of a characteristic Cys loop F/YPxD motif, and degapped (GapStreeze v2.1.0, 25% tolerance) to enable construction of Maximum Likelihood phylogenetic trees (LG model with 500 bootstrap replicates). Homology based searches were also performed to confirm these analyses. TBLASTN searches were performed querying predicted GABA_A_R protein sequences against the genomic sequences of parasitic flatworms (E-value cut off 1e-5, WormBase ParaSite). Gene product identification numbers for all sequences are provided in S1 Table.

### Chemicals

A complete list of chemical structures is provided in S2 Table. Compounds were either sourced commercially (Toronto Research Chemicals (Meclonazepam) and Sigma Aldrich (clonazepam, nitrazepam, diazepam, bromazepam, flurazepam, lorazepam, flunitrazepam)) or synthesized by the Cook Lab. Compounds synthesized by the Cook lab were chosen for screening on schistosomes based on prior studies identifying GABA_A_R-sparing benzodiazepines, reported in references [23–26]. Structures were generated in ChemDraw (v17.1) and clustered by physiochemical properties using ChemMine [27]. Detailed synthesis methods for MCLZ analogs MYM-I-88, MYM-I-91A and MYM-II-53 are provided in S1 File.

### Adult schistosome mobility assays

Female Swiss Webster mice infected with *S*. *mansoni* cercariae (NMRI strain) were sacrificed 49 days post infection by CO_2_ euthanasia. Adult schistosomes were recovered by dissection of the mesenteric vasculature. Harvested schistosomes were washed in DMEM (ThermoFisher cat. # 11995123) supplemented with HEPES (25mM), 5% v/v heat inactivated FCS (Sigma Aldrich cat. # 12133C) and Penicillin-Streptomycin (100 units/mL). Worms were cultured in 6 well dishes (4–5 male worms in 3mL media per well) in the presence of various test compounds or DMSO vehicle control overnight (37°C / 5% CO_2_). Worms were imaged the next day to record movement phenotypes using a Zeiss Discovery v20 stereomicroscope and a QiCAM 12-bit cooled color CCD camera controlled by Metamorph imaging software (version 7.8.1.0). 1 minute recordings were acquired at 4 frames per second and saved as a .TIFF stack, which was imported into ImageJ for analysis. An outline of the workflow used to quantify movement from these video recordings is shown in S1 Fig. Maximum intensity projections were generated for the entire stack of images (241 frames for a 1-minute recording) and integrated pixel values were measured for the resulting composite image, allowing normalization of movement relative to DMSO control treated worms. Inhibition of movement IC_50_ values were calculated using GraphPad Prism v8.1.1 and are expressed ± 95% confidence intervals. Data represents mean ± standard error for ≥3 independent experiments. Significance (*) was determined by unpaired t-test at a threshold of 0.05.

### Transmission electron microscopy

Adult worms were harvested and recovered as above. Fixation was carried out overnight at 4°C in 2.5% glutaraldehyde/2% paraformaldehyde in 0.1 M sodium cacodylate (pH 7.3). Worms were washed 3 × 10 minutes in 0.1 M sodium cacodylate and post-fixed for 2 hours on ice in reduced 1% osmium tetraoxide. Worms were then washed 2 × 10 minutes in distilled water and stained overnight at 4°C in alcoholic Uranyl Acetate. Worms were rinsed in distilled water, dehydrated in 50%, 75% and 95% MeOH, followed by successive 10 minute rinses in 100% MeOH and acetonitrile. Worms were incubated in a 1:1 mix of acetonitrile and epoxy resin for 1 hour prior to 2 × 1 hour incubations in epoxy resin. Worms were then cut transversely and embedded overnight in epoxy resin (60°C). Ultra-thin sections (70 nm) were cut onto bare 200-mesh copper grids and stained in aqueous lead citrate for 1 minute. Sections were imaged on a Hitachi H-600 electron microscope fitted with a Hamamatsu C4742-95 digital camera) operating at an accelerating voltage of 75 kV.

### Binding assays

Binding assays for benzodiazepines against mammalian GABA_A_Rs were performed measuring displacement of [3H] flunitrazepam (0.4 nM) from crude brain membrane preparations [28]. Rat cerebral cortex membrane homogenate (80 μg protein) was incubated with 0.4 nM [3H]-flunitrazepam in 50 mM Tris-HCl (pH 7.7), plus test compounds (screened at concentrations ranging from 0.1 nM to 10 μM), and non-specific binding was assessed by incubation with diazepam (3 μM). Following incubation for 60 min at 4°C, samples were vacuum filtered through glass fiber filters (GF/B, Packard) presoaked with 0.3% PEI and rinsed with ice-cold 50 mM Tris-HCl (Unifilter, Packard). Filters were dried and counted for radioactivity in a scintillation counter (Topcount, Packard). *K*_*i*_ values of test compounds were calculated by the Cheng-Prusoff equation.

### GABA_A_R modeling

Benzodiazepines were docked to the human GABA_A_R cryo-EM structure (PDB ID 6HUO)[29] using Schrödinger Maestro suite v2019-1. Three dimensional ligand structures were generated using the LigPrep module. Water molecules further than 5 Å from the protein surface were removed, hydrogen bonds were optimized at pH 7.0 and the structure was minimized in the OPLS3e forcefield. Ligands were docked into a 10×10×10 Å grid centered on the bound alprazolam molecule in the GABA_A_R structure using Glide [30] in ExtraPrecision (XP) mode, and output poses were ranked by XP GlideScore. Figures were generated using PyMOL 2.3.0.

## Results

### GABA_A_Rs are not the schistosome targets of meclonazepam

The benzodiazepine meclonazepam (MCLZ) is an effective anti-schistosomal drug, but the sedative side effects of MCLZ coincide with the anti-parasitic dose [15]. The sedative side effects of MCLZ are likely driven by human GABA_A_Rs–specifically those heteromeric receptors that contain the α1 subunit [19]. These receptors account for approximately 60% - 80% of brain GABA_A_Rs [19, 31]. But what is the parasite target of MCLZ? From the earliest reports of anti-schistosomal activity of MCLZ , it has been noted that these effects are not replicated by other benzodiazepines that also bind GABA_A_Rs with high affinity [14]. Therefore, we considered that the parasite target of MCLZ may be distinct from GABA_A_Rs, since it is not even clear whether flatworms possess this class of ligand-gated ion channel (LGIC) [32, 33].

**Fig 1:**
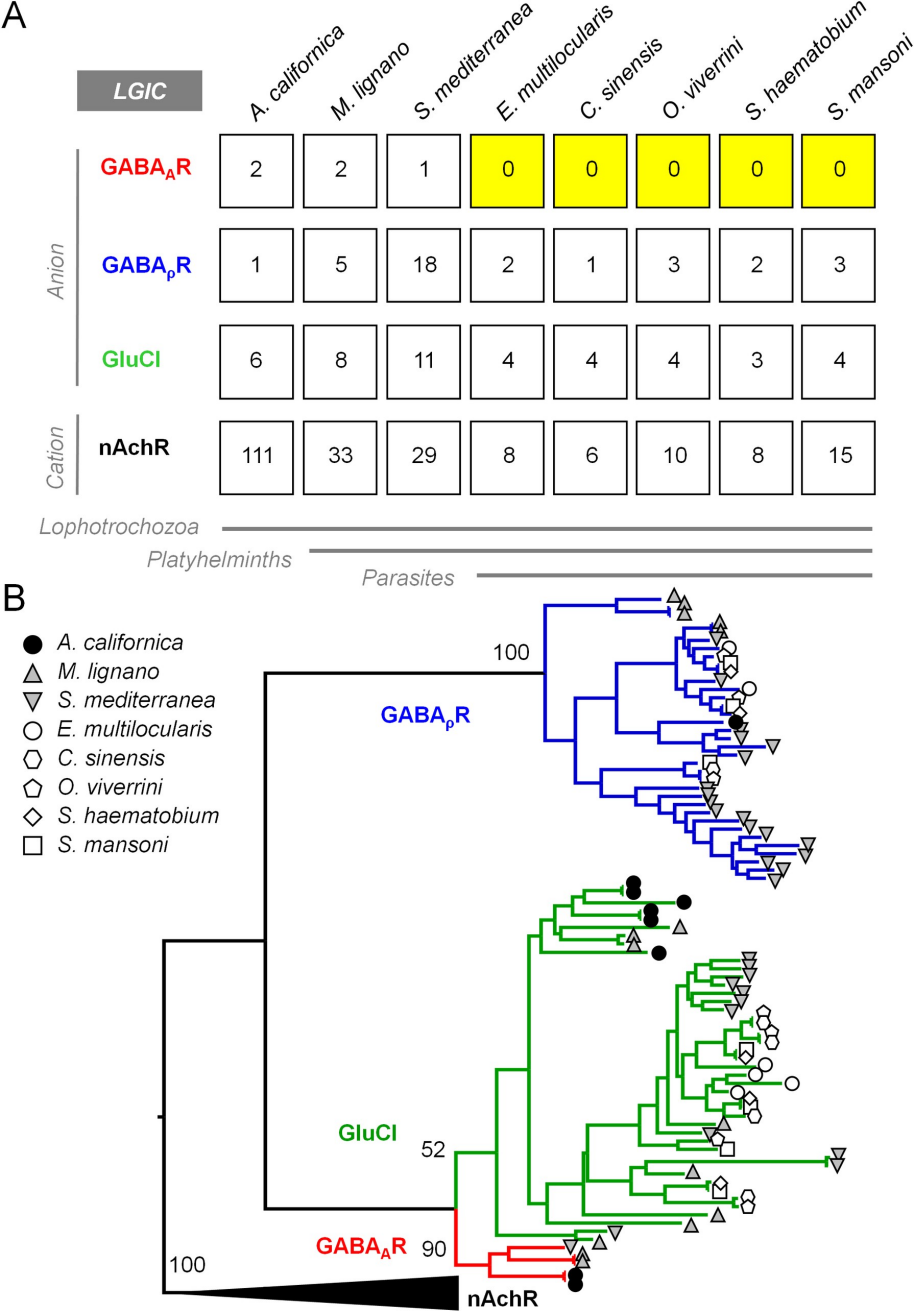
Comparison of free-living and parasitic flatworm Cys-Loop Ligand Gated Ion Channels (LGICs). **(A)** Cys-loop LGICs were curated from the genomes of a non-flatworm lophotrochozoan (*A*. *californica*) as well as free-living (*M*. *lignano*, *S*. *mediterranea*) and parasitic flatworms (*E*. *multilocularis*, *C*. *sinensis*, *O*. *viverrini*, *S*. *haematobium*, *S*. *mansoni*) and clustered into various families of GABA_A_R-like, GABA_ρ_R-like, GluCl-like and nAchR-like sequences. For gene ID numbers, see S1 Table. **(B)** Phylogeny of flatworm and lophotrochozoan LGICs, with a clade of GABA_A_R subunits (red), GABA_ρ_R subunits (blue) and GluCl subunits (green) that include a previously reported flatworm-specific group of receptors [32]. Bootstrap percentage is shown at nodes (500 replicates).

In order to comprehensively search the repertoire of flatworm LGICs, we generated hidden Markov probability models (HMMER v3.2.1) using LGICs curated from a diversity of organisms (humans, *D*. *melanogaster*, *C*. *elegans*, *A*. *californica*). These were used to search the predicted proteomes of various free-living *(S*. *mediterranea*, *M*. *lignano*) and parasitic (*S*. *mansoni*, *S*. *haematobium*, *C*. *sinensis*, *O*. *viverrini*, *E*. *multilocularis*) flatworms to retrieve putative LGICs. Candidates were manually inspected for accurate number of predicted transmembrane domains and the presence of a characteristic Cys-loop F/YPxD motif. The resulting sequences clustered into four groups corresponding to three groups of ligand gated anion channels (Glutamate-gated Chloride Channels (GluCl), GABA_A_Rs and GABA_ρ_Rs) and one group of ligand gated cation channel-like gene products (nicotinic acetylcholine receptor (nAchR)-like) (Fig 1A, S1 Table). Previously reported schistosome GluCls [32] and cholinergic receptors [34] clustered alongside sequences consistent with their functional characterization. GABA_A_Rs are clearly present in the mollusk *A*. *californica* and free-living flatworms *M*. *lignano* and *S*. *mediterranea*, but they appear to have been lost in the parasitic trematode (*S*. *mansoni*, *S*. *haematobium*, *C*. *sinensis*, *O*. *viverrini*) and cestode (*E*. *multilocularis*) species analyzed (Fig 1A & 1B). To confirm that GABA_A_R sequences were not being overlooked due to partial or fragmented gene models, homology-based searches were also performed. TBLASTN search of *A*. *californica* GABA_A_Rs against the genomes of parasitic flatworms found to lack GABA_A_Rs by our HMMER search did not identify additional sequences that clustered with GABA_A_Rs.

### Non-sedating imidazobenzodiazepines cause parasite contractile paralysis

**Fig 2:**
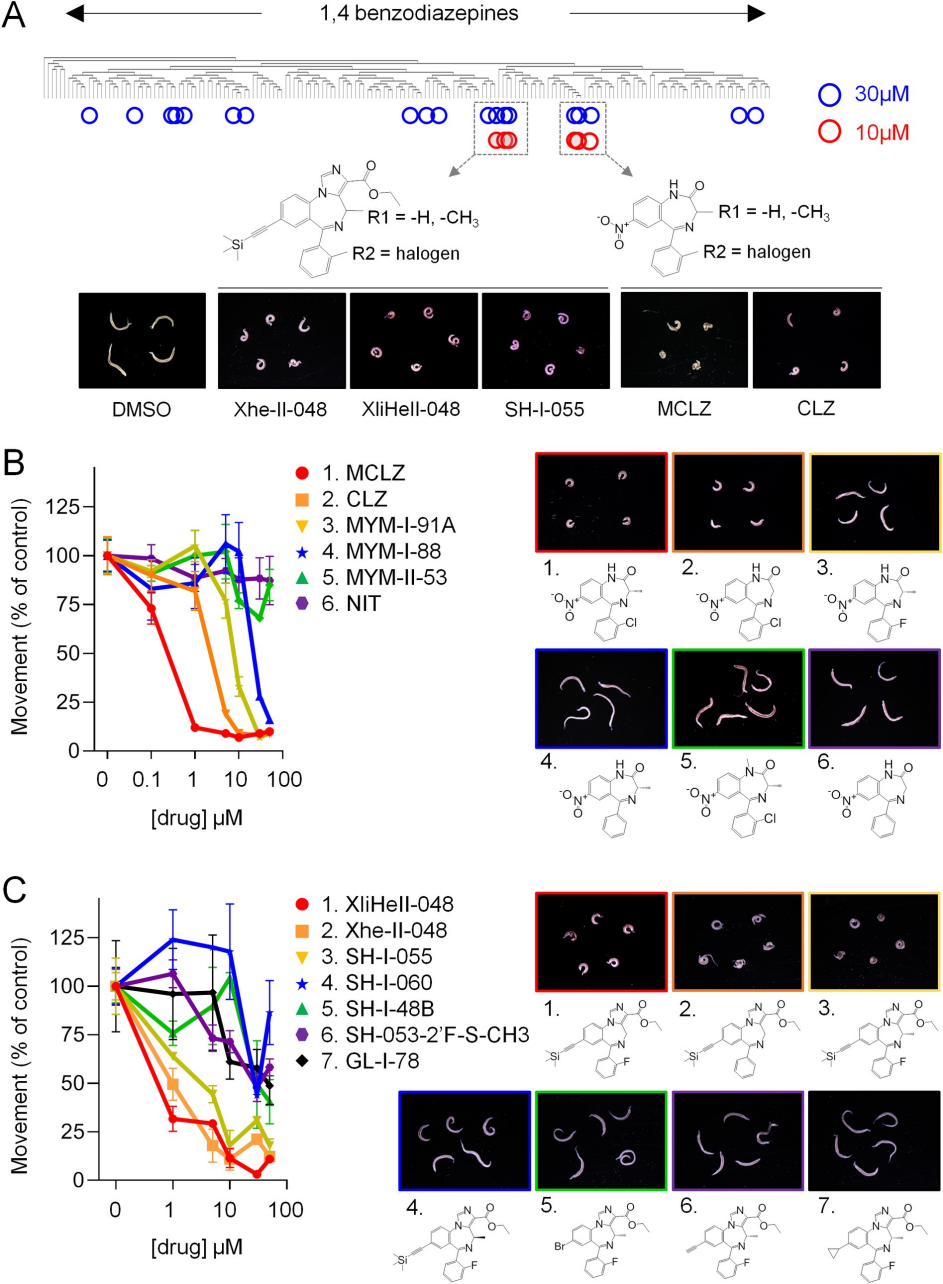
Identification of benzodiazepines with *in vitro* activity against *S*. *mansoni*. **(A)** 180 benzodiazepines were screened for ability to contract and paralyze schistosomes *in vitro*. Compounds were initially screened at 30 μM (hits = blue circles), and active compounds were re-screened at 10 μM (hits = red circles). This prioritized imidazobenzodiazepines with a TMS-acetylene moiety (left) and meclonazepam-like compounds (right). Structure-activity relationship of **(B)** a series of MCLZ derivatives and **(C)** a series of imidazobenzodiazepines. MCLZ = meclonazepam, CLZ = clonazepam, NIT = nitrazepam. Left = movement concentration-response curves for parasites exposed to each compound. Right = images of drug treated worms (10 μM, overnight).

If schistosomes lack GABA_A_Rs, then the parasite target of MCLZ may have different structural requirements for ligand binding than the MCLZ:GABA_A_R interaction that drives sedation. If so, then it should be possible to identify parasite-selective benzodiazepines that retain anti-schistosomal activity but lack affinity for mammalian GABA_A_Rs that contain α1-subunits. Therefore, we screened a library of compounds that included various α1GABA_A_R-sparing compounds against adult male *S*. *mansoni* and assessed action on schistosomes *in vitro*. Worms were harvested from mice 7-weeks post-infection and cultured in test compound (30 μM) overnight, after which video recordings were acquired to measure effects on worm movement relative to vehicle negative control (DMSO 0.1% v/v) and MCLZ (5 μM) positive control. This primary screen of 180 compounds identified 19 ligands that phenocopied MCLZ, exhibiting coiled, contractile phenotype and paralysis (Fig 2A). Active compounds were then re-screened at 10 μM to refine the hits to the most active compounds. This resulted in the prioritization of two chemical series. The first were MCLZ derivatives, including clonazepam (CLZ, lacking the C3 methyl group of MCLZ), and the derivative MYM-I-91A with the phenyl C2’ halogen substituted from a chlorine to a fluorine (Fig 2A & 2B). The second series was a group of imidazobenzodiazepines (SH-I-055, XliHeII-048 and XHE-II-048) that also varied in the C3 and phenyl C2’ positions (Fig 2A & 2C).

The structure activity relationship of these two series was explored, assessing the comparative potency of various derivatives at impairing schistosome movement across a series of doses. Qualitative observations on the activity of various MCLZ-like benzodiazepines have been previously reported [14, 35, 36], and the activity of the MCLZ-like series was largely consistent with these studies. The rank order of potency was MCLZ (IC_50_ 160 nM), CLZ (IC_50_ 1.9 μM), MYM-I-91A (IC_50_ 7.2 μM), MYM-I-88 (IC_50_ 26.0 μM), followed by MYM-II-53 and nitrazepam which were inactive at concentrations as high as 50 μM (Fig 2B). The structure-activity relationship of the imidazobenzodiazepines was explored, assessing the ability of the three hit compounds and structurally related, less active compounds to evoke coiled, contractile paralysis (Fig 2C). The most active compounds, XliHeII-048 (IC_50_ 540 nM) and Xhe-II-048 (IC_50_ 850 nM), contain an imidazole-ester group at the benzodiazepine N1-C2 position and a trimethylsilyl (TMS) acetylene group at the C7 position. The two compounds differ only by the addition of a fluorine at the phenyl C2’ position on XliHeII-048. A third compound, SH-I-055 (IC_50_ 1.4 μM) was identical to XliHeII-048 except for the addition of a chiral (S)-methyl group at the C3 position. When this chiral methyl group was in the (R) orientation there was a marked decrease in potency (compound SH-I-060). Finally, compounds retaining SH-I-055’s (S)-methyl group but with varying C7 modifications in place of the TMS acetylene group all showed dramatically decreased affinity (GL-I-78, SH-I-48B and SH-053-2’F-S-CH3 with a cyclopropyl, bromine and alkyne group, respectively).

**Fig 3:**
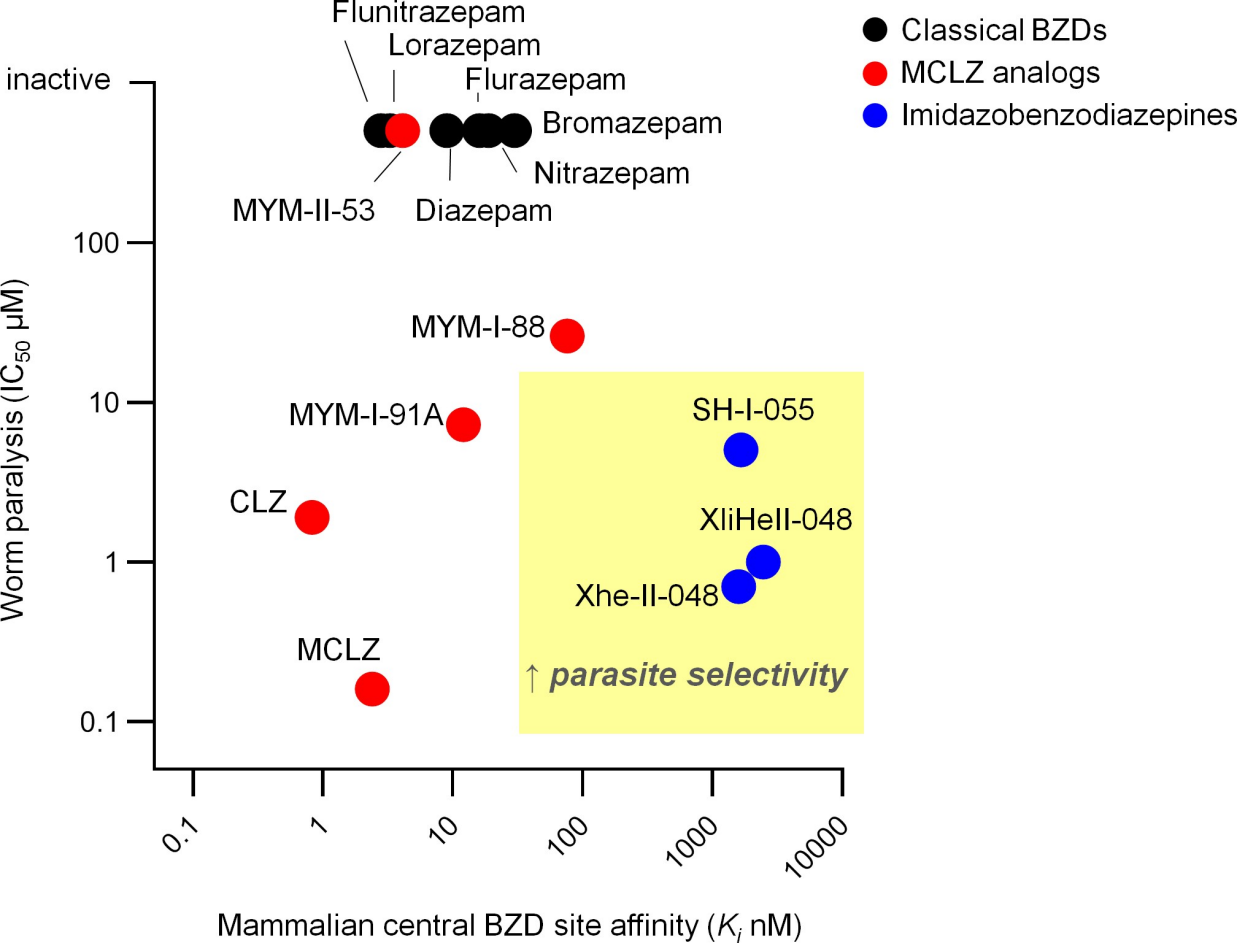
Relative mammalian GABA_A_R affinity and schistosome potency of various benzodiazepines. Scatter plot of mammalian central benzodiazepine binding site affinity (*K*_*i*_) versus schistosome activity (movement IC_50_). Sedating compounds active against worms (i.e. MCLZ) fall within the lower left quadrant. Desired compounds that lack sedation but retain anti-parasitic effects fall within the lower right quadrant. Red = MCLZ analogs. Blue = imidazobenzodiazepines. Black = classical benzodiazepines. Mammalian central benzodiazepine binding site *K*_*i*_ values for flunitrazepam, lorazepam, diazepam, flurazepam, nitrazepam, and bromazepam are from reference [37].

The potency of XliHeII-048 and Xhe-II-048 at inhibiting worm movement was comparable to the active ligands in the MCLZ derivative series with IC_50_ values in the high nanomolar range (Fig 2B & 2C). Therefore, binding assays were performed to compare the relative affinities of these two chemical series for mammalian GABA_A_Rs (Fig 3). As expected, MCLZ potently displaced [3H]-flunitrazepam from rat brain membrane preparations (*K*_*i*_ = 2.4 nM). The related compound CLZ bound GABA_A_Rs with an even higher affinity (*K*_*i*_ = 0.82 nM). Analogs MYM-I-91A and MYM-II-53 also displayed high affinity (*K*_*i*_ = 12.1 nM and 4.1 nM, respectively). MYM-I-88, which lacks a halogen on the phenyl ring, displayed markedly reduced binding (*K*_*i*_ = 76.4 nM). The imidazobenzodiazepine series displayed a GABA_A_R affinity roughly three logs less potent than MCLZ (Xhe-II-048 *K*_*i*_ = 2.5 μM, SH-I-055 *K*_*i*_ = 1.7 μM, XliHeII-048 *K*_*i*_ = 1.6 μM).

### Imidazobenzodiazepine Xhe-II-048 causes structural damage to parasite tissue

**Fig 4:**
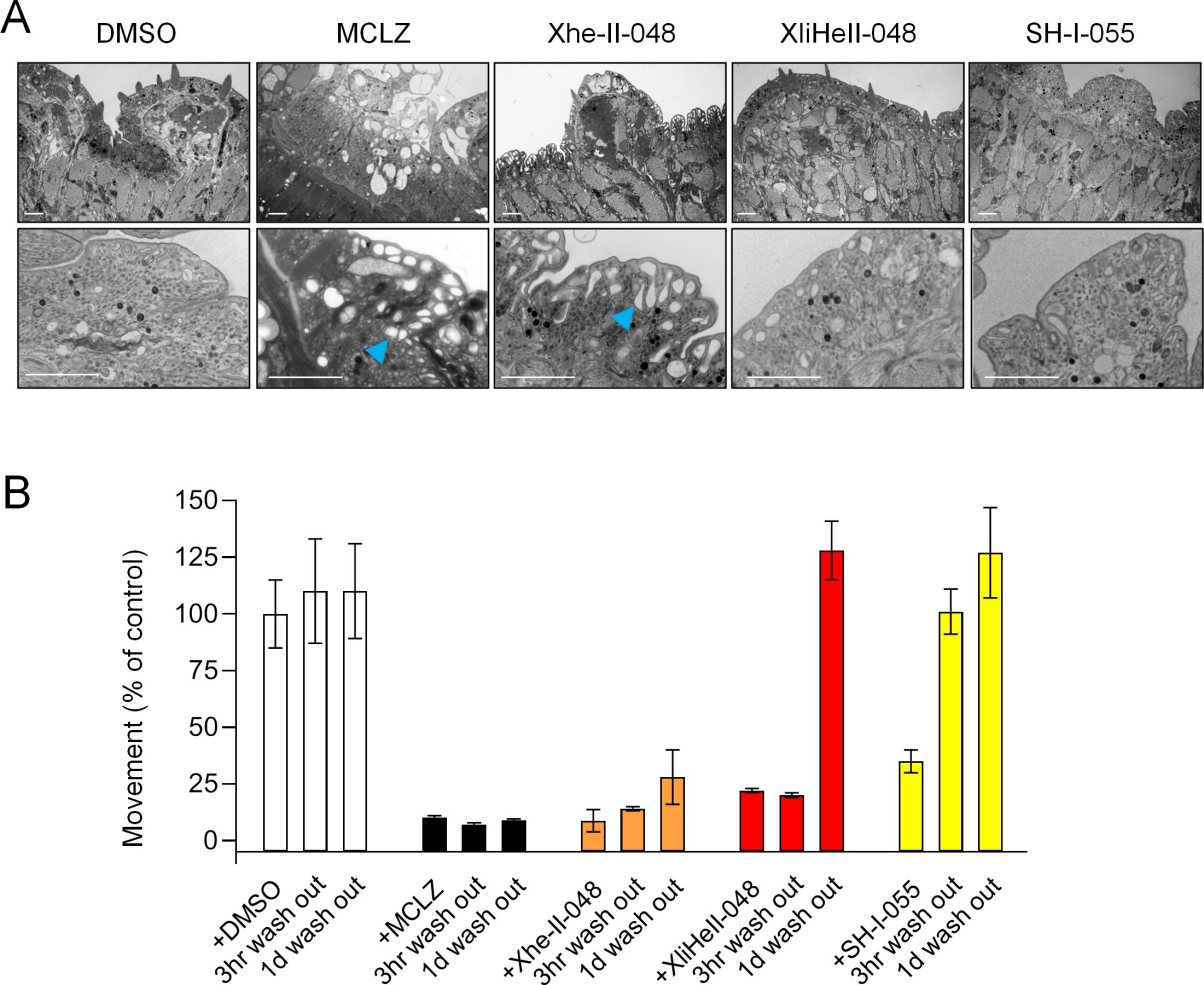
Xhe-II-048 damages the schistosome tegument. **(A)** Transmission electron microscopy images of transverse sections of *S*. *mansoni* exposed to either DMSO control, MCLZ (5 μM) or various imidazobenzodiazepines (10 μM, 14 hours). Dorsal tegument is oriented to the top. Scale = 2 μm. Arrowed = vacuoles beneath the tegument apical membrane. **(B)** Movement of worms treated with DMSO, MCLZ, or TMS-acetylene imidazobenzodiazepine compounds (10 μM, 14 hours), and recovery at various timepoints (3 hours, 1 day) following drug washout.

Given the parasite-selectivity of imidazobenzodiazepines (Fig 3, blue) relative to MCLZ-like compounds (Fig 3, red), we investigated the effects of XliHeII-048, Xhe-II-048 and SH-I-055 on schistosome tissues in more detail. Specifically, we were interested in drug-evoked damage to the parasite tegument, which is a feature of many anti-schistosomal compounds [38]. Worms treated with DMSO vehicle control, MCLZ (5 μM) or various imidazobenzodiazepines (10 μM, based on effective concentrations determined in Fig 2) overnight were fixed and processed for imaging by transmission electron microcopy (TEM). Imaging transverse schistosome cross sections revealed a typical body wall structure in DMSO treated worms, with alternating layers of schistosome muscle, followed by the tegument basal membrane, tegument syncytium, and tegument apical membrane. In MCLZ treated worms, tissue layers are disrupted, with pervasive vacuolization of the tegument (Fig 4A). The tegument of Xhe-II-048 treated worms displayed a similar pattern of extensive vacuole distribution beneath the apical membrane, while worms treated with XliHeII-048 and the less potent imidazobenzodiazepine SH-I-055 displayed normal tissue ultrastructure (Fig 4A).

MCLZ is a schistocidal compound, and worms do not recover movement following drug washout (Fig 4B). Similarly, Xhe-II-048 treated worms did not recover movement up to one day following drug washout, consistent with the extensive ultrastructural damage caused by this compound (Fig 4A). Compounds XliHeII-048 and SH-I-055, which did not cause pervasive tegument damage, evoked only a transient paralysis, recovering by 1 day after drug washout.

## Discussion

The anti-schistosomal activity of the benzodiazepine meclonazepam (MCLZ) was discovered in the 1970’s, but development of this lead as a human therapy for schistosomiasis stalled due to sedative side effects. While MCLZ was capable of curing infection, the doses required coincided with the onset of sedative side effects [14, 15]. This is expected, given the structural similarity of MCLZ to centrally acting benzodiazepines such as CLZ and NIT that are clinically used as anxiolytics and display well known sedating effects. Some attempts were made in the 1980’s to antagonize the sedative effects of MCLZ using the GABA_A_R antagonist flumazenil. While flumazenil did not impair the anti-schistosomal effect of MCLZ [39], pharmacokinetic differences between flumazenil (administered by IV due to poor bioavailability, <1 hour elimination half-life [40]) and MCLZ (orally bioavailable, half-life up to 80 hours[41]) precluded the development of an admixture as a viable non-sedating therapeutic approach [16, 42]. Consequently, research on MCLZ as an anti-schistosomal lead has slowed over the past several decades. However, we have revisited this compound based on recent helminth genomic data indicating that parasitic flatworms lack GABA_A_Rs (Fig 1), advances in our understanding of mammalian GABA_A_R subtypes that account for sedative side effects [19, 43], and advances in the development of non-sedating benzodiazepines with selectivity towards various GABA_A_R sub-types [23–26].

### Schistosome genomes lack GABA_A_Rs

Sequenced genomes of parasitic trematode (*S*. *mansoni*, *S*. *haematobium*, *C*. *sinensis*, *O*. *viverrini*) and cestode (*E*. *multilocularis*) flatworms lack GABA_A_Rs, the benzodiazepine targets that cause sedation (Fig 1). Gene loss is common with the evolution of parasitism [44, 45], and a lack of GABA_A_Rs is consistent with prior bioinformatic characterization of parasitic flatworm LGICs [21, 33, 46].

If parasitic flatworms lack GABA_A_Rs, might MCLZ act on related cys-loop ligand-gated ion channels (GluCls and GABAρRs)? Experimental and bioinformatic evidence indicates that this is unlikely. MCLZ is inactive against SmGluCl, a representative of the class of flatworm chloride channels most similar to GABA_A_Rs [32]. Recent structural data resolving the interactions of classical benzodiazepines with human GABA_A_Rs provide an explanation for this [29]. Specifically, the human α1His102 residue that interacts with the benzodiazepine C7 position at the interface of the GABA_A_R α1 and γ subunits is replaced by an arginine in the flatworm-specific GluCls. This position is important, since human α4 and α6 GABA_A_R subunits also contain an arginine in this position, and the larger sidechain likely sterically clashes with classical benzodiazepines to render GABA_A_Rs comprised of α4 and α6 subunits benzodiazepine-insensitive. In the case of each of the three schistosome GABAρRs, the α1His102 position contains a negatively charged aspartic acid. This switch from a positively charged histidine sidechain may oppose interactions with the electron-dense benzodiazepine C7 position. The inactivity of benzodiazepines on flatworm cys-loop LGICs is consistent with the observation that, aside from MCLZ, and to a lesser degree the structurally similar compound CLZ, benzodiazepines lack anti-schistosomal activity ([14], Fig 3, S2 Table). These findings support the hypothesis that the parasite target is distinct from the human GABA_A_Rs that account for dose-limiting sedation. However, the possibility that the parasite receptor of MCLZ may have structural similarity to mammalian GABA_A_Rs (even if it lacks sequence similarity) cannot be excluded until this target is deorphanized.

Resolution of the schistosome target of MCLZ is a high priority, as this may enable the design of ligands with broader anti-parasitic activity. While MCLZ is active against the two major African species of schistosomes, it is inactive against the Asian schistosome *S*. *japonicum*. This situation is similar to the example of the anti-schistosomal drug oxamniquine. Oxamniquine is only effective against *S*. *mansoni*, but discovery of the parasite drug target has ultimately enabled the design of analogs with broad range activity against all three of the major schistosome species (*S*. *mansoni*, *S*. *japonicum* and *S*. *haematobium*)[47, 48].

### *S*. *mansoni* are paralyzed by a class of α1GABA_A_R-sparing benzodiazepines

**Fig 5:**
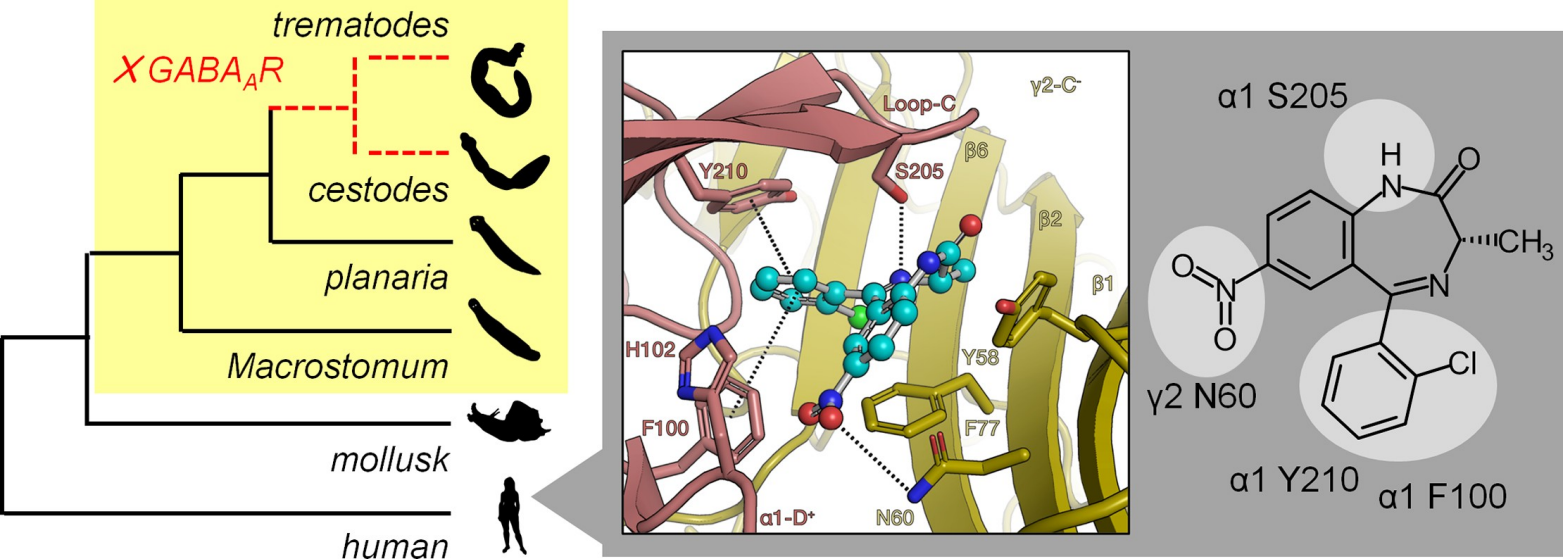
MCLZ interaction with host GABA_A_Rs. Within the flatworm phylum (highlighted yellow), GABA_A_Rs are present in free living Macrostomum and planarians, but are lost in parasitic flatworms (dashed line). *Inset*—While the flatworm target has still to be identified, *in silico* docking of MCLZ to the benzodiazepine binding site of the solved human heteromeric α1β3γ2 GABA_A_R cryo-EM structure [29] reveals predicted interactions with amino acid side chains on the α1 and γ2 subunits (dashed lines). *Right* - 2D chemical structure of MCLZ, with potential GABA_A_R interactions highlighted.

While benzodiazepines are typically considered GABA_A_R ligands with anxiolytic or sedative properties, numerous α1GABA_A_R-sparing members of this class have been developed with indications as diverse as anti-asthma to anti-viral medications [25, 49, 50]. Several imidazobenzodiazepines with a C7 trimethylsilyl (TMS) acetylene group caused schistosome contractile paralysis *in vitro* at high nanomolar to low micromolar concentrations (Fig 2A & 2C) and display low GABA_A_R binding affinity (Fig 3). These compounds were originally synthesized as part of a chemical series exploring α2 / α3-selective benzodiazepines as potential anxiolytic, anticonvulsant and antinociceptive leads [24, 26]. The differing GABA_A_R affinities of MCLZ and the imidazobenzodiazepine hits may be due to the interaction of the MCLZ N1 position with the α1 S205, which is disrupted by the addition of the imidazole ring. Modification of this N1 is also observed in the non-sedating antiviral benzodiazepine BDAA [50], although smaller alkyl groups are likely tolerated, such as the methyl on diazepam and MYM-II-53. Additionally, the large TMS-acetylene group at the C7 position of the imidazobenzodiazepine hit series may not be tolerated within the GABA_A_R binding pocket, where the MCLZ C7 nitro group is predicted to interact with the γ N60 sidechain (Fig 5).

While these ligands phenocopy MCLZ to a degree, it is unclear whether they act via the same schistosome receptor—or if they do bind the same receptor, whether they share a common binding pose. The target of MCLZ will need to be identified to generate hypotheses into ligand-receptor structure-activity relationships (SAR), as the structures of MCLZ and the TMS-acetylene imidazobenzodiazepines appear quite different. However, there are similarities and differences in the SAR of the two series.

Two interesting positions are (i) the benzodiazepine C3 position, which is typically unmodified in classical benzodiazepines but contains a chiral methyl group in these two series, and (ii) the phenyl C2’ position, which is commonly halogenated in benzodiazepines with GABA_A_R affinity. The benzodiazepine C3 position is essential for MCLZ activity, the key difference between clonazepam and MCLZ is that clonazepam lacks a C3 methyl group. This results in a roughly >3 log decrease in potency (Fig 3, [51]). Chirality of this position is also important for 3-methylclonazepam, since the (R) enantiomer reportedly exhibits reduced anti-parasitic efficacy [36]. On the other hand, C3 methylation decreased activity of XliHeII-048. The (S)-methylated compound SH-I-055 was ~3 times less potent than XliHeII-048, and the (R)-methylated compound SH-I-060 was essentially inactive. This schistosome SAR is distinct from the binding of imidazobenzodiazepines to mammalian GABA_A_Rs, where (S) and (R) isomers have roughly equivalent binding affinities [52]. The halogen on the phenyl C2’ position of MCLZ seems important for schistosome activity, given that nitrazepam (inactive against parasites) differs from clonazepam (movement IC_50_ 1.9 μM) in that the phenyl ring is unsubstituted. However, XliHeII-048 (which contains a fluorine at this position) and Xhe-II-048 (which possesses an unsubstituted phenyl ring) appear equipotent (Fig 2C). In fact, the unsubstituted compound Xhe-II-048 was unique in evoking structural damage to the parasite tegument (Fig 4A).

Development of the Xhe-II-048 hit compound into a bona fide anti-schistosomal lead will likely require modifications to improve metabolic stability *in vivo*. Specifically, the ester and TMS-acetylene groups will likely require substitution with bioisosteres. The imidazole ester group is likely rapidly hydrolyzed by carboxylesterases during first pass metabolism, and the TMS-acetylene is also unlikely to be stable *in vivo* [53]. From the SAR shown in Fig 2C, it is apparent that loss of the TMS-acetylene group dramatically decreased potency. Nevertheless, this is the first report of non-sedating benzodiazepines screened against schistosomes. MCLZ has anti-schistosomal activity in human clinical trials, but with an extremely narrow therapeutic index; the effective anti-parasitic dose (0.2–0.3 mg/kg) coincided with the dose at which sedation was reported (above 0.3 mg/kg) [15]. Here, we have identified benzodiazepine hits that exhibit potent anti-schistosomal effects *in vitro* and dramatically lower affinity for host α1GABA_A_Rs (Fig 3). Given the scarcity of new lead compounds to treat schistosomiasis, these data are valuable in advancing a pharmacophore that retains anti-schistosomal activity while displaying reduced sedation.

## Supporting information

S1 Fig Quantification of schistosome movement. Worm movement was quantified from video recordings (1 minute duration, 4 frames per second). **(A)** Video recordings in color (i) were converted to gray scale and inverted so that worms were transformed to dark silhouettes against a light background (ii). Video recordings (.tiff stacks of 241 images) were treated as a Z-stack, with a composite image of the maximum intensity from each frame integrated into one composite image (iii). **(B)** Movement was quantified by calculating the pixel intensity values of the drug treated composite and expressed relative to the DMSO vehicle control treated composite, producing a numerical quantification of movement across each concentration.
(TIF)

S1 Table List of putative Aplysia and flatworm LGICs. Sequence IDs reflect putative LGICs curated from *A*. *californica* (all NCBI deposited proteins for taxonomy ID 6500), *S*. *mediterranea* (assembly ASM260089v1), *M*. *lignano* (assembly Mlig_3_7), *E*. *multilocularis* (assembly EMULTI002), *Clonorchis sinensis* (assembly ASM360417v1), *O*. *viverrini* (assembly OpiViv1.0), *S*. *haematobium* (assembly SchHae_1.0) and *S*. *mansoni* (assembly v7) and clustered into either nAch, GluCl, GABA_A_ or GABA_ρ_ like receptors. Other than *A*. *californica*, all assemblies were accessed via WormBase ParaSite.
(XLSX)

S2 Table Structures and phenotypes of benzodiazepines screened against *S*. *mansoni*. Data from the primary screen shown in Fig 2A. SMILES IDs are provided for all compounds screened, and phenotypes are shown for worms exposed to 30 μM test compound overnight. Compounds highlighted in blue active at 30 μM and in red at 10 μM.
(XLSX)

S1 File Detailed methods for the synthesis of MCLZ analogs. Methods for the synthesis of MCLZ derivatives with modifications to the N1 position and phenyl C2’ position.
(DOCX)

## References

[1] Hotez PJ, Fenwick A. Schistosomiasis in Africa: an emerging tragedy in our new global health decade. PLoS NTD. 2009;3(9):e485 Epub 2009/09/30. 10.1371/journal.pntd.0000485 19787054 PMC2746322

[2] LoVerde PT. Schistosomiasis. Adv Exp Med Biol. 2019;1154:45–70. Epub 2019/07/13. 10.1007/978-3-030-18616-6_3 . 31297759

[3] Disease GBD, Injury I, Prevalence C. Global, regional, and national incidence, prevalence, and years lived with disability for 328 diseases and injuries for 195 countries, 1990–2016: a systematic analysis for the Global Burden of Disease Study 2016. Lancet. 2017;390(10100):1211–59. Epub 2017/09/19. 10.1016/S0140-6736(17)32154-2 28919117 PMC5605509

[4] King CH, Dangerfield-Cha M. The unacknowledged impact of chronic schistosomiasis. Chronic illness. 2008;4(1):65–79. Epub 2008/03/07. 10.1177/1742395307084407 . 18322031

[5] Andrews P, Thomas H, Pohlke R, Seubert J. Praziquantel. Med Res Rev. 1983;3(2):147–200. Epub 1983/04/01. 10.1002/med.2610030204 . 6408323

[6] Olliaro PL, Vaillant MT, Belizario VJ, Lwambo NJ, Ouldabdallahi M, Pieri OS, et al A multicentre randomized controlled trial of the efficacy and safety of single-dose praziquantel at 40 mg/kg vs. 60 mg/kg for treating intestinal schistosomiasis in the Philippines, Mauritania, Tanzania and Brazil. PLoS Negl Trop Dis. 2011;5(6):e1165 Epub 2011/06/23. 10.1371/journal.pntd.0001165 21695161 PMC3114749

[7] Coulibaly JT, Panic G, Silue KD, Kovac J, Hattendorf J, Keiser J. Efficacy and safety of praziquantel in preschool-aged and school-aged children infected with Schistosoma mansoni: a randomised controlled, parallel-group, dose-ranging, phase 2 trial. Lancet Glob Health. 2017;5(7):e688–e98. Epub 2017/06/18. 10.1016/S2214-109X(17)30187-0 28619227 PMC5471607

[8] Ismail M, Metwally A, Farghaly A, Bruce J, Tao LF, Bennett JL. Characterization of isolates of Schistosoma mansoni from Egyptian villagers that tolerate high doses of praziquantel. Am J Trop Med Hyg. 1996;55(2):214–8. Epub 1996/08/01. 10.4269/ajtmh.1996.55.214 . 8780463

[9] Kron M, Gordon C, Bauers T, Lu Z, Mahatme S, Shah J, et al Persistence of Schistosoma japonicum DNA in a Kidney-Liver Transplant Recipient. Am J Trop Med Hyg. 2019;100(3):584–7. Epub 2019/01/11. 10.4269/ajtmh.18-0752 30628570 PMC6402933

[10] Jesudoss Chelladurai J, Kifleyohannes T, Scott J, Brewer MT. Praziquantel Resistance in the Zoonotic Cestode Dipylidium caninum. Am J Trop Med Hyg. 2018;99(5):1201–5. Epub 2018/09/19. 10.4269/ajtmh.18-0533 30226153 PMC6221203

[11] Gryseels B, Mbaye A, De Vlas SJ, Stelma FF, Guisse F, Van Lieshout L, et al Are poor responses to praziquantel for the treatment of Schistosoma mansoni infections in Senegal due to resistance? An overview of the evidence. Trop Med Int Health. 2001;6(11):864–73. Epub 2001/11/13. 10.1046/j.1365-3156.2001.00811.x . 11703840

[12] Sabah AA, Fletcher C, Webbe G, Doenhoff MJ. *Schistosoma mansoni*: chemotherapy of infections of different ages. Exp Parasitol. 1986;61(3):294–303. 10.1016/0014-4894(86)90184-0 . 3086114

[13] Bergquist R, Utzinger J, Keiser J. Controlling schistosomiasis with praziquantel: How much longer without a viable alternative? Infect Dis Poverty. 2017;6(1):74 10.1186/s40249-017-0286-2 28351414 PMC5371198

[14] Stohler H. Ro 11–3128, a novel schistosomicidal compound. Proceedings of the 10th International Congress of Chemotherapy. 1978;1:147–8.

[15] Baard AP, Sommers DK, Honiball PJ, Fourie ED, du Toit LE. Preliminary results in human schistosomiasis with Ro 11–3128. S Afr Med J. 1979;55(16):617–8. . 380021

[16] Darragh A, Lambe R, Brick I, Downie WW. Reversal of benzodiazepine-induced sedation by intravenous Ro 15–1788. Lancet. 1981;2(8254):1042 Epub 1981/11/07. 10.1016/s0140-6736(81)91233-2 . 6118493

[17] Darragh A, Lambe R, Brick I, O'Boyle C. Antagonism of the central effects of 3-methylclonazepam. Br J Clin Pharmacol. 1982;14(6):871–2. Epub 1982/12/01. 10.1111/j.1365-2125.1982.tb02052.x 6817773 PMC1427549

[18] O'Boyle C, Lambe R, Darragh A. Central effects in man of the novel schistosomicidal benzodiazepine meclonazepam. Eur J Clin Pharmacol. 1985;29(1):105–8. Epub 1985/01/01. 10.1007/bf00547377 . 4054198

[19] McKernan RM, Rosahl TW, Reynolds DS, Sur C, Wafford KA, Atack JR, et al Sedative but not anxiolytic properties of benzodiazepines are mediated by the GABA(A) receptor alpha1 subtype. Nat Neurosci. 2000;3(6):587–92. Epub 2000/05/18. 10.1038/75761 . 10816315

[20] Rudolph U, Knoflach F. Beyond classical benzodiazepines: novel therapeutic potential of GABAA receptor subtypes. Nature reviews Drug discovery. 2011;10(9):685–97. Epub 2011/07/30. 10.1038/nrd3502 21799515 PMC3375401

[21] International Helminth Genomes Consortium. Comparative genomics of the major parasitic worms. Nat Genet. 2019;51(1):163–74. Epub 2018/11/07. 10.1038/s41588-018-0262-1 30397333 PMC6349046

[22] Tsirigos KD, Peters C, Shu N, Kall L, Elofsson A. The TOPCONS web server for consensus prediction of membrane protein topology and signal peptides. Nucleic Acids Res. 2015;43(W1):W401–7. Epub 2015/05/15. 10.1093/nar/gkv485 25969446 PMC4489233

[23] Clayton T, Chen JL, Ernst M, Richter L, Cromer BA, Morton CJ, et al An updated unified pharmacophore model of the benzodiazepine binding site on gamma-aminobutyric acid(a) receptors: correlation with comparative models. Curr Med Chem. 2007;14(26):2755–75. Epub 2007/11/30. 10.2174/092986707782360097 . 18045122

[24] Huang Q, He X, Ma C, Liu R, Yu S, Dayer CA, et al Pharmacophore/receptor models for GABA(A)/BzR subtypes (alpha1beta3gamma2, alpha5beta3gamma2, and alpha6beta3gamma2) via a comprehensive ligand-mapping approach. J Med Chem. 2000;43(1):71–95. Epub 2000/01/14. 10.1021/jm990341r . 10633039

[25] Gallos G, Yocum GT, Siviski ME, Yim PD, Fu XW, Poe MM, et al Selective targeting of the alpha5-subunit of GABAA receptors relaxes airway smooth muscle and inhibits cellular calcium handling. Am J Physiol Lung Cell Mol Physiol. 2015;308(9):L931–42. Epub 2015/02/11. 10.1152/ajplung.00107.2014 25659897 PMC4421780

[26] He X, Huang Q, Ma C, Yu S, McKernan R, Cook JM. Pharmacophore/receptor models for GABA(A)/BzR alpha2beta3gamma2, alpha3beta3gamma2 and alpha4beta3gamma2 recombinant subtypes. Included volume analysis and comparison to alpha1beta3gamma2, alpha5beta3gamma2, and alpha6beta3gamma2 subtypes. Drug Des Discov. 2000;17(2):131–71. Epub 2000/10/25. . 11045902

[27] Backman TW, Cao Y, Girke T. ChemMine tools: an online service for analyzing and clustering small molecules. Nucleic Acids Res. 2011;39(Web Server issue):W486–91. Epub 2011/05/18. 10.1093/nar/gkr320 21576229 PMC3125754

[28] Speth RC, Wastek GJ, Yamamura HI. Benzodiazepine receptors: temperature dependence of [3H]flunitrazepam binding. Life Sci. 1979;24(4):351–7. Epub 1979/01/22. 10.1016/0024-3205(79)90331-x . 34765

[29] Masiulis S, Desai R, Uchanski T, Serna Martin I, Laverty D, Karia D, et al GABAA receptor signalling mechanisms revealed by structural pharmacology. Nature. 2019;565(7740):454–9. Epub 2019/01/04. 10.1038/s41586-018-0832-5 30602790 PMC6370056

[30] Friesner RA, Banks JL, Murphy RB, Halgren TA, Klicic JJ, Mainz DT, et al Glide: a new approach for rapid, accurate docking and scoring. 1. Method and assessment of docking accuracy. J Med Chem. 2004;47(7):1739–49. Epub 2004/03/19. 10.1021/jm0306430 . 15027865

[31] Benke D, Fakitsas P, Roggenmoser C, Michel C, Rudolph U, Mohler H. Analysis of the presence and abundance of GABAA receptors containing two different types of alpha subunits in murine brain using point-mutated alpha subunits. J Biol Chem. 2004;279(42):43654–60. Epub 2004/08/12. 10.1074/jbc.M407154200 . 15304513

[32] Dufour V, Beech RN, Wever C, Dent JA, Geary TG. Molecular cloning and characterization of novel glutamate-gated chloride channel subunits from Schistosoma mansoni. PLoS pathogens. 2013;9(8):e1003586 Epub 2013/09/07. 10.1371/journal.ppat.1003586 24009509 PMC3757052

[33] Berriman M, Haas BJ, LoVerde PT, Wilson RA, Dillon GP, Cerqueira GC, et al The genome of the blood fluke *Schistosoma mansoni*. Nature. 2009;460:352–60. 10.1038/nature08160 19606141 PMC2756445

[34] MacDonald K, Buxton S, Kimber MJ, Day TA, Robertson AP, Ribeiro P. Functional characterization of a novel family of acetylcholine-gated chloride channels in *Schistosoma mansoni*. PLoS pathogens. 2014;10(6):e1004181 10.1371/journal.ppat.1004181 24945827 PMC4055736

[35] Bennett JL. Characteristics of antischistosomal benzodiazepine binding sites in Schistosoma mansoni. J Parasitol. 1980;66(5):742–7. . 7007599

[36] Szente A, inventor; Hoffmann-La Roche Inc, assignee. Benzodiazepine derivatives1977 10/14/1975.

[37] Braestrup C, Squires RF. Pharmacological characterization of benzodiazepine receptors in the brain. Eur J Pharmacol. 1978;48(3):263–70. Epub 1978/04/01. 10.1016/0014-2999(78)90085-7 . 639854

[38] Bricker CS, Depenbusch JW, Bennett JL, Thompson DP. The Relationship between Tegumental Disruption and Muscle-Contraction in *Schistosoma mansoni* Exposed to Various Compounds. Zeitschrift Fur Parasitenkunde-Parasitology Research. 1983;69(1):61–71. ISI:A1983QC59200007. 10.1007/BF00934011 6837102

[39] Hunkeler W, Mohler H, Pieri L, Polc P, Bonetti EP, Cumin R, et al Selective antagonists of benzodiazepines. Nature. 1981;290(5806):514–6. Epub 1981/04/09. 10.1038/290514a0 . 6261143

[40] Roncari G, Ziegler WH, Guentert TW. Pharmacokinetics of the new benzodiazepine antagonist Ro 15–1788 in man following intravenous and oral administration. Br J Clin Pharmacol. 1986;22(4):421–8. Epub 1986/10/01. 10.1111/j.1365-2125.1986.tb02912.x 3094572 PMC1401170

[41] Coassolo P, Aubert C, Cano JP. Plasma determination of 3-methylclonazepam by capillary gas chromatography. J Chromatogr. 1985;338(2):347–55. Epub 1985/03/22. 10.1016/0378-4347(85)80105-5 . 3998022

[42] Darragh A, Lambe R, Scully M, Brick I, O'Boyle C, Downe WW. Investigation in man of the efficacy of a benzodiazepine antagonist, Ro 15–1788. Lancet. 1981;2(8236):8–10. Epub 1981/07/04. 10.1016/s0140-6736(81)90251-8 . 6113428

[43] Rowlett JK, Platt DM, Lelas S, Atack JR, Dawson GR. Different GABAA receptor subtypes mediate the anxiolytic, abuse-related, and motor effects of benzodiazepine-like drugs in primates. Proc Natl Acad Sci U S A. 2005;102(3):915–20. Epub 2005/01/13. 10.1073/pnas.0405621102 15644443 PMC545524

[44] Jackson AP. The evolution of parasite genomes and the origins of parasitism. Parasitology. 2015;142 Suppl 1:S1–5. Epub 2015/02/07. 10.1017/S0031182014001516 25656359 PMC4413782

[45] Tsai IJ, Zarowiecki M, Holroyd N, Garciarrubio A, Sanchez-Flores A, Brooks KL, et al The genomes of four tapeworm species reveal adaptations to parasitism. Nature. 2013;496(7443):57–63. 10.1038/nature12031 23485966 PMC3964345

[46] Young ND, Jex AR, Li B, Liu S, Yang L, Xiong Z, et al Whole-genome sequence of *Schistosoma haematobium*. Nat Genet. 2012;44(2):221–5. Epub 2012/01/17. 10.1038/ng.1065 . 22246508

[47] Rugel A, Tarpley RS, Lopez A, Menard T, Guzman MA, Taylor AB, et al Design, Synthesis, and Characterization of Novel Small Molecules as Broad Range Antischistosomal Agents. ACS medicinal chemistry letters. 2018;9(10):967–73. Epub 2018/10/23. 10.1021/acsmedchemlett.8b00257 30344901 PMC6187409

[48] Valentim CL, Cioli D, Chevalier FD, Cao X, Taylor AB, Holloway SP, et al Genetic and molecular basis of drug resistance and species-specific drug action in schistosome parasites. Science. 2013;342(6164):1385–9. Epub 2013/11/23. 10.1126/science.1243106 24263136 PMC4136436

[49] Carter MC, Alber DG, Baxter RC, Bithell SK, Budworth J, Chubb A, et al 1,4-Benzodiazepines as inhibitors of respiratory syncytial virus. J Med Chem. 2006;49(7):2311–9. Epub 2006/03/31. 10.1021/jm051185t . 16570927

[50] Guo F, Wu S, Julander J, Ma J, Zhang X, Kulp J, et al A Novel Benzodiazepine Compound Inhibits Yellow Fever Virus Infection by Specifically Targeting NS4 B Protein. J Virol. 2016;90(23):10774–88. Epub 2016/09/23. 10.1128/JVI.01253-16 27654301 PMC5110185

[51] Menezes CM, Rivera G, Alves MA, do Amaral DN, Thibaut JP, Noel F, et al Synthesis, biological evaluation, and structure-activity relationship of clonazepam, meclonazepam, and 1,4-benzodiazepine compounds with schistosomicidal activity. Chem Biol Drug Des. 2012;79(6):943–9. Epub 2012/02/11. 10.1111/j.1747-0285.2012.01354.x . 22321778

[52] Elgarf AA, Siebert DCB, Steudle F, Draxler A, Li G, Huang S, et al Different Benzodiazepines Bind with Distinct Binding Modes to GABAA Receptors. ACS Chem Biol. 2018;13(8):2033–9. Epub 2018/05/17. 10.1021/acschembio.8b00144 29767950 PMC6102643

[53] Wuts TWGaPGM. Protection for the Alkyne–CH In: Wuts TWGaPGM, editor. Protective Groups in Organic Synthesis: John Wiley & Sons, Inc.; 1999 p. 654–9.

