# Supplementary figures and images for: Non-sedating benzodiazepines cause paralysis and tissue damage in the parasitic blood fluke Schistosoma mansoni
Source: PLoS Negl Trop Dis. 2019 Nov 15;13(11):e0007826. doi: 10.1371/journal.pntd.0007826 (PMC6881066; doi:10.1371/journal.pntd.0007826)

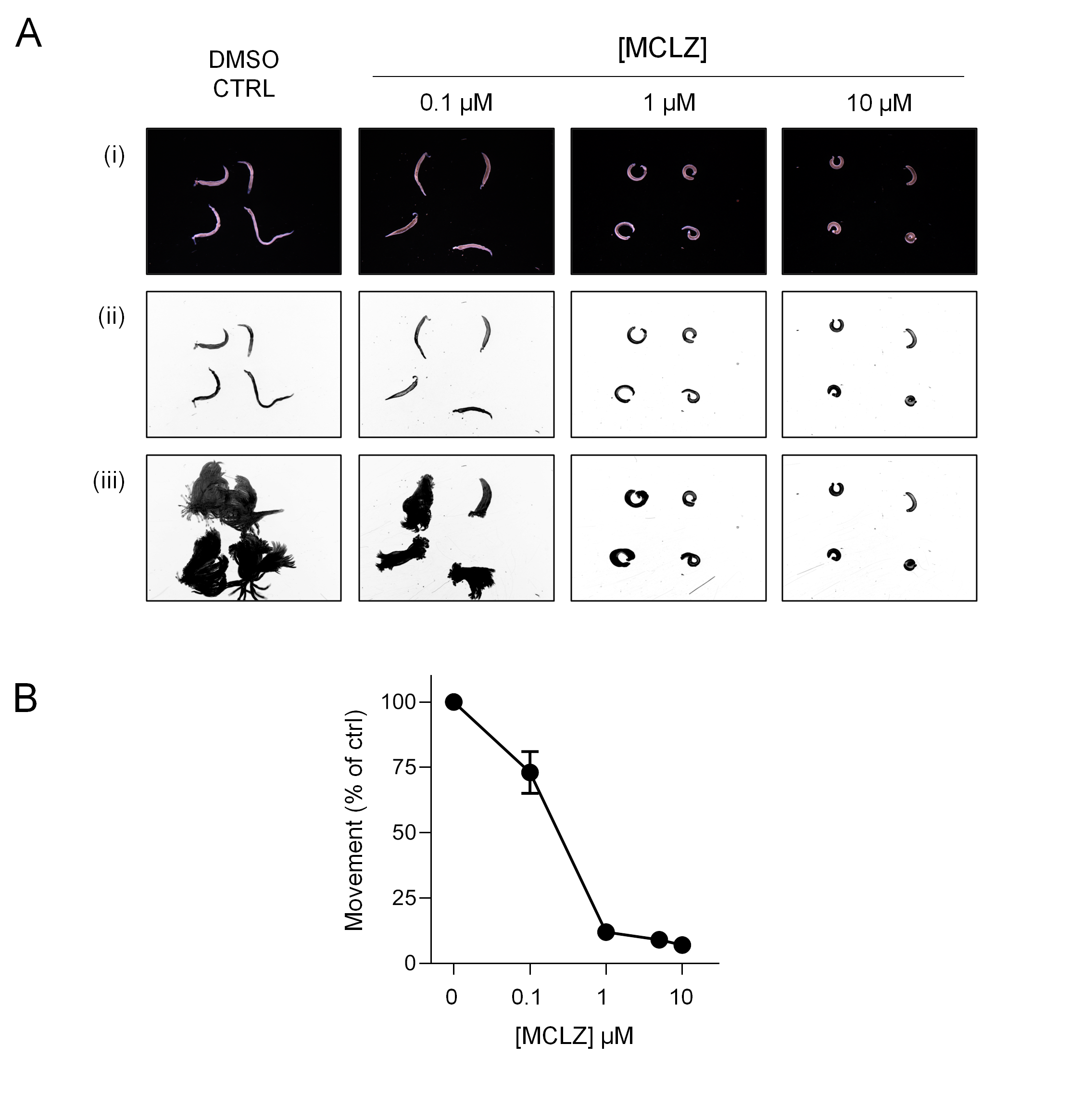

Supplement: S1 Fig — Worm movement was quantified from video recordings (1 minute duration, 4 frames per second). (A) Video recordings in color (i) were converted to gray scale and inverted so that worms were transformed to dark silhouettes against a light background (ii). Video recordings (.tiff stacks of 241 images) were treated as a Z-stack, with a composite image of the maximum intensity from each frame integrated into one composite image (iii). (B) Movement was quantified by calculating the pixel intensity values of the drug treated composite and expressed relative to the DMSO vehicle control treated composite, producing a numerical quantification of movement across each concentration. (TIF) [file pntd.0007826.s001.tif]
