# Supplementary material for: Non-sedating benzodiazepines cause paralysis and tissue damage in the parasitic blood fluke Schistosoma mansoni
Source: PLoS Negl Trop Dis. 2019 Nov 15;13(11):e0007826. doi: 10.1371/journal.pntd.0007826 (PMC6881066; doi:10.1371/journal.pntd.0007826)
Supplement: S1 File — Methods for the synthesis of MCLZ derivatives with modifications to the N1 position and phenyl C2’ position. (DOCX) [file pntd.0007826.s004.docx]

**Synthesis of MCLZ analogs MYM-I-88, MYM-I-91A and MYM-II-53**

(*S*)-3-Methyl-7-nitro-5-phenyl-1,3-dihydro-2H-benzo[e][1,4]diazepin-2-one (**4**); The starting amide **1**^1-2^ (2 g, 7.99 mmol) was dissolved in acetic acid (4 mL) and this solution was poured into a round bottom flask containing sulfuric acid (8 mL) at 0 ^°^C. The resulting mixture was stirred for 10 min until homogenous and then a solution of nitric acid (0.5 mL, 11.985 mmol) and sulfuric acid (3 mL) was added to the mixture at -10 ^°^C. This solution was then stirred at 25 ^°^C for 2.5 h. The reaction mixture was then poured into a flask of water contacting ice (200 g). The mixture was brought to pH 7-8 by addition of 25% aq. potassium carbonate. It was then extracted with ethyl acetate (3 × 25 mL). The combined organic layer was washed with 25% aq. potassium carbonate (1 × 20 mL) and brine (3 × 20 mL) and then dried (MgSO_4_). The solvent was removed under reduced pressure and the residue was purified by column chromatography (neutral alumina, EtOAc: hexane, 3:2) to afford pure nitro intermediate **4** as a brown solid (1.2 g, 51%). ^1^H NMR (500 MHz, CDCl_3_) δ 9.89 (s, 1H, CONH), 8.39 (dd, *J* = 8.9, 2.6 Hz, 1H, Ar), 8.26 (d, *J* = 2.5 Hz, 1H, Ar), 7.53 – 7.48 (m, 3H, Ar), 7.44 – 7.36 (m, 3H, Ar), 3.81 (d, *J* = 6.4 Hz, 1H, CH), 1.82 (d, *J* = 6.4 Hz, 3H, CH_3_). ^13^C NMR (126 MHz, CDCl_3_) δ 172.59 (s), 167.74 (s), 143.36 (s), 142.71 (s), 138.08 (s), 131.06 (s), 129.66 (s, 2C), 128.66 (s, 2C), 127.63 (s), 127.10 (s), 126.42 (s), 121.97 (s), 59.16 (s), 16.96 (s).

(*S*)-5-(2-Fluorophenyl)-3-methyl-7-nitro-1,3-dihydro-2H-benzo[e][1,4]diazepin-2-one (**5**); The starting 2’-F-S-CH_3_ isomer **2**^1-2^ (500 mg, 1.864 mmol) was dissolved in acetic acid (3 mL) and this solution was poured into a round bottom flask at 0 ^°^C containing sulfuric acid (4 mL). The mixture was stirred for 10 min until homogeneous and then a solution of nitric acid (0.112 mL, 2.796 mmol) and sulfuric acid (2 mL) was added to the solution at -10 ^°^C. This mixture was then stirred at 25 ^°^C for 2.5 h. The reaction mixture was then poured into a flask containing ice (100 g). The mixture which resulted was brought to pH 7-8 with 25% aq. potassium carbonate and then extracted with ethyl acetate (3 × 15 mL). The combined organic layer was then washed with 25% aq. potassium carbonate (1 × 10 mL), brine (3 × 10 mL) and dried (MgSO_4_). The solvent was removed under reduced pressure and the residue was purified by column chromatography (neutral alumina, EtOAc: hexane, 3:2) to afford pure 2’-F-7-nitrobenzodiazepine **5** as an off-white solid (0.21 g, 36%). ^1^H NMR (500 MHz, CDCl_3_) δ 9.80 (s, 1H, CONH), 8.36 (dd, *J* = 8.9, 2.4 Hz, 1H, Ar), 8.17 (d, *J* = 1.8 Hz, 1H, Ar), 7.71 (t, *J* = 6.9 Hz, 1H, Ar), 7.52 (dd, *J* = 13.0, 6.2 Hz, 1H, Ar), 7.37 – 7.27 (m, 3H, Ar), 7.08 (t, *J* = 10, 1H, Ar), 3.84 (q, *J* = 6.4 Hz, 1H, CH), 1.82 (d, *J* = 6.5 Hz, 3H, CH_3_). ^13^C NMR (126 MHz, CDCl_3_) δ 172.20 (s), 164.49 (s), 160.42 (d, *J* = 251.6 Hz), 143.18 (s), 142.16 (s), 132.87 (d, *J* = 8.5 Hz), 131.58 (d, *J* = 1.9 Hz), 128.54 (d, *J* = 1.1 Hz), 126.51 (s), 126.42 (s), 125.77 (d, *J* = 1.8 Hz), 124.80 (d, *J* = 3.5 Hz), 122.00 (s), 116.41 (d, *J* = 21.5 Hz), 59.19 (s), 16.88 (s).

(*S*)-5-(2-chlorophenyl)-1,3-dimethyl-7-nitro-1,3-dihydro-2H-benzo[e][1,4]diazepin-2-one (**7):** An oven dried 3 neck round bottom flask was charged with meclonazepam **6** (80 mg, 0.243 mmol) (Anant Pharmaceuticals Pvt. Ltd., India), anhydrous *N*,*N*-dimethyl formamide (5 ml) and methyl iodide (0.02 ml, 0.32 mmol). The mixture was stirred for 10 min until homogeneous and cooled to -30 °C. Then potassium *tert*-butoxide (27 mg, 0.254 mmol) was added to the reaction mixture. The solution was stirred at 25 °C for 2 hours. The reaction was monitored by TLC. After completion the reaction was quenched by adding ice cold water and then extracted with ethyl acetate (3 × 10 mL). The combined organic layer was washed with 25% aq. ammonium chloride (1 × 10 mL), brine (3 × 10 mL) and dried (Na_2_SO_4_). The solvent was removed under reduced pressure and the solid obtained was purified by flash chromatography (neutral alumina, EtOAc: hexane, 3:2) to afford pure methylated meclonazepam **7** as yellow solid (75 mg, 91%). ^1^H NMR (500 MHz, CDCl_3_) δ 8.38 (dd, *J* = 9.1, 2.7 Hz, 1H, Ar), 7.96 (d, *J* = 2.6 Hz, 1H, Ar), 7.71 – 7.65 (m, 1H, Ar), 7.50 (d, *J* = 9.1 Hz, 1H, Ar), 7.47 – 7.43 (m, 2H, Ar), 7.39 – 7.35 (m, 1H, Ar), 3.80 (d, *J* = 6.5 Hz, 1H, CH), 3.55 (s, 3H, N-CH_3_), 1.78 (d, *J* = 6.5 Hz, 3H, CH_3_). ^13^C NMR (126 MHz, CDCl_3_) δ 170.31, 166.62, 147.78, 143.12, 137.22, 133.00, 131.59, 131.27, 130.78, 130.29, 127.47, 126.09, 124.16, 122.06, 59.09, 35.33, 17.28.

1. Arnold, A.E.; Stafford, D.C.; Cook, J.M.; Emala, C.W.; Forkuo, G.; Jahan, R.; Kodali, R.; Li,G.; Stephen, M.R.; International Patent WO 2018/035246 A1, Feb. 22, 2018.

2. Jayasundara, C.R.K.; Unold, J.M.; Oppenheimer, J.; Smith, M.R, III.; Maleckzka, R.E.,Jr.; *Org. Lett.* **2014**, 16, 6072−6075

dx.doi.org/10.1021/ol5028738

3. Strazzolini, P.; Giumanini, A.G.; Runcio, A.; *Tetrahedron Lett*. **2001**, 42, 1387 – 1389

<https://doi.org/10.1016/S0040-4039(00)02253-X>
