# Supplementary material for: Non-sedating benzodiazepines cause paralysis and tissue damage in the parasitic blood fluke Schistosoma mansoni
Source: PLoS Negl Trop Dis. 2019 Nov 15;13(11):e0007826. doi: 10.1371/journal.pntd.0007826 (PMC6881066; doi:10.1371/journal.pntd.0007826)
Supplement: Attachment — Submitted filename: RESPONSE_TO_REVIEWERS.docx [file pntd.0007826.s005.docx]

We are grateful to the reviewers and editor for the thoughtful and thorough review of our manuscript. Our responses to the reviewers’ comments are outlined below:

***Reviewer #1***

***• The methods are appropriate and are clearly presented. My only minor concern is that previous work has been done on BZ binding to membranes; were these methods designed de novo or were they based on earlier, similar work? If the latter is the case, that earlier work should be cited.***

BZ binding assays are performed per established protocols. Reference 28 has been added (line 143).

***• Reviewer #1: The results are clearly and concisely presented and the data are convincing. The authors might note that previous bioinformatics searches had already shown that S. mansoni lacks GABAr sequences.***

Prior bioinformatics analysis of *S. mansoni* are cited (references 21 & 33, line 317). However, neither of these analyses identified GABAρ receptors that we have reported (Smp_334630, Smp_135040, Smp336620). This is in part because of extensive revisions and improvements to the *S. mansoni* genome that have occurred since its publication in 2009 (reference 33), and the different methodology performed in reference 44.

***• Reviewer #1: The conclusions are reasonable and well-supported by the data. The sentence beginning on line 286 is unclear; it seems to indicate that it is expected that the sedative dose of MCLZ is the same as the therapeutic dose for schistosomiasis. Please clarify.***

The indicated text has been modified for clarity (new lines 299-300). Prior human clinical trials indeed showed that the dose at which the sedative side effects of MCLZ manifest is the same as the dose required to cure schistosomiasis (references 15-18).

***Reviewer #2:***

***• Bioinformatics of LGICs: were homology-based searches also used to confirm missing channels? This would help in case fragmented gene models (or missing gene models) did not meet the transmembrane count filter.***

Homology based searches have now been performed. This analysis supports our finding that GABA_A_Rs are not present in parasitic flatworms. See new text lines 98-100 and 191-195.

***• Reviewer #2: Throughout the manuscript, 'selectivity' is profiled by measuring in vitro effects on schistosomes and specific interactions with mammalian GABAAR channels. There is the real possibility that selecting for chemicals that maintain 'parasite-selectivity' and diminished affinity for GABAAR channels is akin to selecting for activity against a different homologous parasite and host target set. Since the mode of action that drives this selectivity is unknown, this possibility cannot yet be ruled out. While not in the scope of this manuscript, I think it would be valuable to devote some text to discussing this possibility.***

New text addressing this possibly has now been added to lines 334-336 of the discussion.

***• Reviewer #2: Differences in recovery among imidazobenzodiazepines (Fig 4) are not well-explained. I would be curious to know whether higher concentrations of XliHe-II-048 and SH-I-055 imidazobenzodiazepines would have prevented recovery - suggesting this is a threshold effect.***

We would expect that worms will be irreversibly effected / killed given a high enough concentration of nearly any compound. The concentration of the imidazobenzodiazepines (10 µM) was chosen for the recovery assays in the basis of concentration-response curves in Figure 2 that showed worms were maximally affected by this concentration of XliHe-II-048, Xhe-II-048 and SH-I-055. MCLZ was used at a lower concentration in these assays, because concentration-response curves demonstrated it was potent. We refrained from exploring concentrations of drug in the high µM range, because while they may produce an *in vitro* phenotype it is unlikely to be relevant *in vivo*. Text has been modified (lines 273-274) to clarify that the concentration of imidazobenzodiazepines selected was based on concentration-response curves in Figure 2.

***Reviewer #3***

***•* Figure 1: The authors show the GABA_A_R class is absent from Schmidtea, Echinococcus and Schistosoma. Given that there are many more flatworm species available in WormBase ParaSite, would it be possible for the authors to screen those for homologs too, to be absolutely sure these genes are not present in some trematodes?**

As suggested, we have expanded our analysis to include other trematodes. Figure 1 and Supplemental Table 1 have been revised to include the *Schistosoma haematobium* and the liver flukes *Clonorchis sinensis* and *Opisthorchis viverrini*. Since the initial submission, a new release of WormBase ParaSite included an improved *Schmidtea mediterranea* genome and so this species was also re-analyzed. This analysis found that free-living *S. mediterranea* and *Macrostomum lignano* flatworms both possess GABA_A_Rs, which are absent in parasitic species.

***•* Figure 2: In figure 2 the authors show 180 benzodiazepines that they screened. It's not clear to me how these compounds were chosen, are they just all benzodiazepines that the authors could get their hands on, or was there some criteria for selecting the compounds? Please could the authors clarify this.**

These 180 compounds were chosen since they have been previously studied for their varied affinity against human GABAARs. Dr. Cook’s lab has spent >40 years synthesizing various benzodiazepines to investigate the structure-activity relationship of these compounds at human GABA_A_R. This prior work (references 23-26 and citations therein) identified structures that lack affinity for the α1GABA_A_Rs that cause sedation. Various derivatives of meclonazepam were also synthesized (Figure 2B), given the proven efficacy of this compound. Text clarifying the rationale for the selection of these compounds is now included (lines 106-108).

***•* Figure 4: the authors show what happens with MCLZ at 5 microM but Xhe-II-048 at 10 microM. I may have missed it, but is there no effect with Xhe-II-048 at 5 microM? Also, I am wondering have the authors tested Xhe-II-048 on other species such as S. haematobium and tapeworms to see if there is an effect on tegument of other flatworm species, which might suggest it would act as a drug for those species too?**

The choice of imidazobenzodiazepine concentrations for experiments in Figure 4 is addressed above in response to reviewer 2. We agree with reviewer 3 that the efficacy of these benzodiazepines against other parasites is a fascinating question. The long-term goal is of course to identify a broad-spectrum anthelmintic (see next point). As of now we do not the resources to maintain additional animal models and life-cycles in our lab to test this series, but this is the logical direction for future work to explore.

***•* Discussion: It would be of great interest to find a drug that can be used for all the different species of schistosome, for example, for Schistosoma haematobium and S. japonicum also. Have the authors tested or considered testing the imidazobenzodiazepines against other schistosome species (and even other flatworm parasites like Fasciola and Echinococcus)? I presume that this will be a next step for the authors, in addition to tests on different stages of schistosomes (somules, juveniles), and in vivo studies with these drugs in mice? I would like the authors to add some discussion on what they consider the most important next step to develop these compounds as new drugs.**

Reviewer 3 is correct that the future direction of this work will involve exploring the efficacy of these leads against other flatworm parasites. In addition to designing new derivatives with improved *in vivo* stability to enable *in vivo* mouse studies, we consider identification of the parasite target of MCLZ to be a high priority for future work. A similar example in the literature is the anti-schistosomal drug oxamniquine, which is only efficacious against *S. mansoni.* However, identification of the parasite target has enabled the rational design of new analogs with a broad anti-parasitic profile against all three major schistosome species. New text has been added in the discussion elaborating on these future directions (lines 338-344).
